# Exploring Braak’s Hypothesis of Parkinson’s Disease

**DOI:** 10.3389/fneur.2017.00037

**Published:** 2017-02-13

**Authors:** Carmen D. Rietdijk, Paula Perez-Pardo, Johan Garssen, Richard J. A. van Wezel, Aletta D. Kraneveld

**Affiliations:** ^1^Division of Pharmacology, Faculty of Science, Utrecht Institute for Pharmaceutical Sciences, Utrecht University, Utrecht, Netherlands; ^2^Nutricia Research, Utrecht, Netherlands; ^3^Department of Biomedical Signals and Systems, MIRA, University of Twente, Enschede, Netherlands; ^4^Department of Biophysics, Donders Institute for Brain, Cognition and Behaviour, Radboud University Nijmegen, Nijmegen, Netherlands

**Keywords:** Parkinson’s disease, Braak’s hypothesis, Lewy pathology, αSynuclein, enteric nervous system

## Abstract

Parkinson’s disease (PD) is a neurodegenerative disorder for which there is no cure. Most patients suffer from sporadic PD, which is likely caused by a combination of genetic and environmental factors. Braak’s hypothesis states that sporadic PD is caused by a pathogen that enters the body *via* the nasal cavity, and subsequently is swallowed and reaches the gut, initiating Lewy pathology (LP) in the nose and the digestive tract. A staging system describing the spread of LP from the peripheral to the central nervous system was also postulated by the same research group. There has been criticism to Braak’s hypothesis, in part because not all patients follow the proposed staging system. Here, we review literature that either supports or criticizes Braak’s hypothesis, focused on the enteric route, digestive problems in patients, the spread of LP on a tissue and a cellular level, and the toxicity of the protein αSynuclein (αSyn), which is the major constituent of LP. We conclude that Braak’s hypothesis is supported by *in vitro, in vivo*, and clinical evidence. However, we also conclude that the staging system of Braak only describes a specific subset of patients with young onset and long duration of the disease.

## Introduction

Parkinson’s disease (PD) is an incurable neurodegenerative disease hallmarked by damage to the dopaminergic neurons of the substantia nigra (SN), and αSynuclein (αSyn) containing inclusion bodies (Lewy pathology; LP) in the surviving neurons, resulting in characteristic motor impairment. The prevalence of PD in Europe ranges between 65.6 and 12,500 per 100,000, and the annual incidence rate ranges between 5 and 346 per 100,000 (1). The variation in these prevalence and incidence rates could be due to genetic or environmental factors, differences in case ascertainment or diagnostic criteria, or different age distributions in the populations (countries) studied (1). In the US population of 65 years and older, PD is more common in Caucasians and Hispanics, than Afro-Americans and Asians (2, 3), indicating a genetic factor may be (partially) responsible for the differences found in the European study. Current treatments for PD include medicinal treatment using levodopa (4, 5), and surgical treatment using deep brain stimulation (6). Although these treatments offer relief of symptoms, they do not cure the disease. All in all, it is clear that PD is an important neurodegenerative disorder to study, even with the more conservative estimations of prevalence and incidence, since currently no cure or preventative treatment exists.

There are two forms of PD: familial and sporadic. The familial form is caused by genetic aberrations, among others in the gene for αSyn [point mutations A30P (7), A53T (8), E46K (9), H50Q (10, 11), and G51D (12), or locus duplication (13, 14) or triplication (15, 16)]. The cause for sporadic PD is not known, but some progress has been made in the search for potential causes, implicating both genetic and environmental factors. The pesticides rotenone and paraquat (17), and the toxin MPTP (18) (1-methyl-4-fenyl-1,2,3,6-tetrahydropyridine; a toxic byproduct of the opioid analgesic desmethylprodine, MPPP, a synthetic heroin), are known to cause PD in humans, explaining some cases of sporadic PD. Additionally, two twin studies have found that sporadic PD has a significant genetic component (19, 20). As mentioned above, in the US, a difference was found in the incidence and prevalence of PD between the Caucasian and Hispanic versus Afro-American and Asian population, also showing a genetic influence (2). On the other hand, a recent review by Pan-Montojo and Reichmann suggests an important role of toxic environmental substances in the etiology of sporadic PD (21). Although the exact influence of genetic and environmental factors in sporadic PD is not known, some elements of disease development have been identified, most importantly neuroinflammation, oxidative stress, and αSyn misfolding and aggregation (22–29). Misfolding and aggregation of αSyn is suspected to lead to LP in surviving neurons, and thus combatting αSyn aggregation has been suggested to be of potential therapeutic value (30). It seems likely that both environmental and genetic factors interact to cause sporadic PD. As a result, the search for potential environmental factors has been ongoing in PD research.

## Braak’s Hypothesis

**Figure 1:**
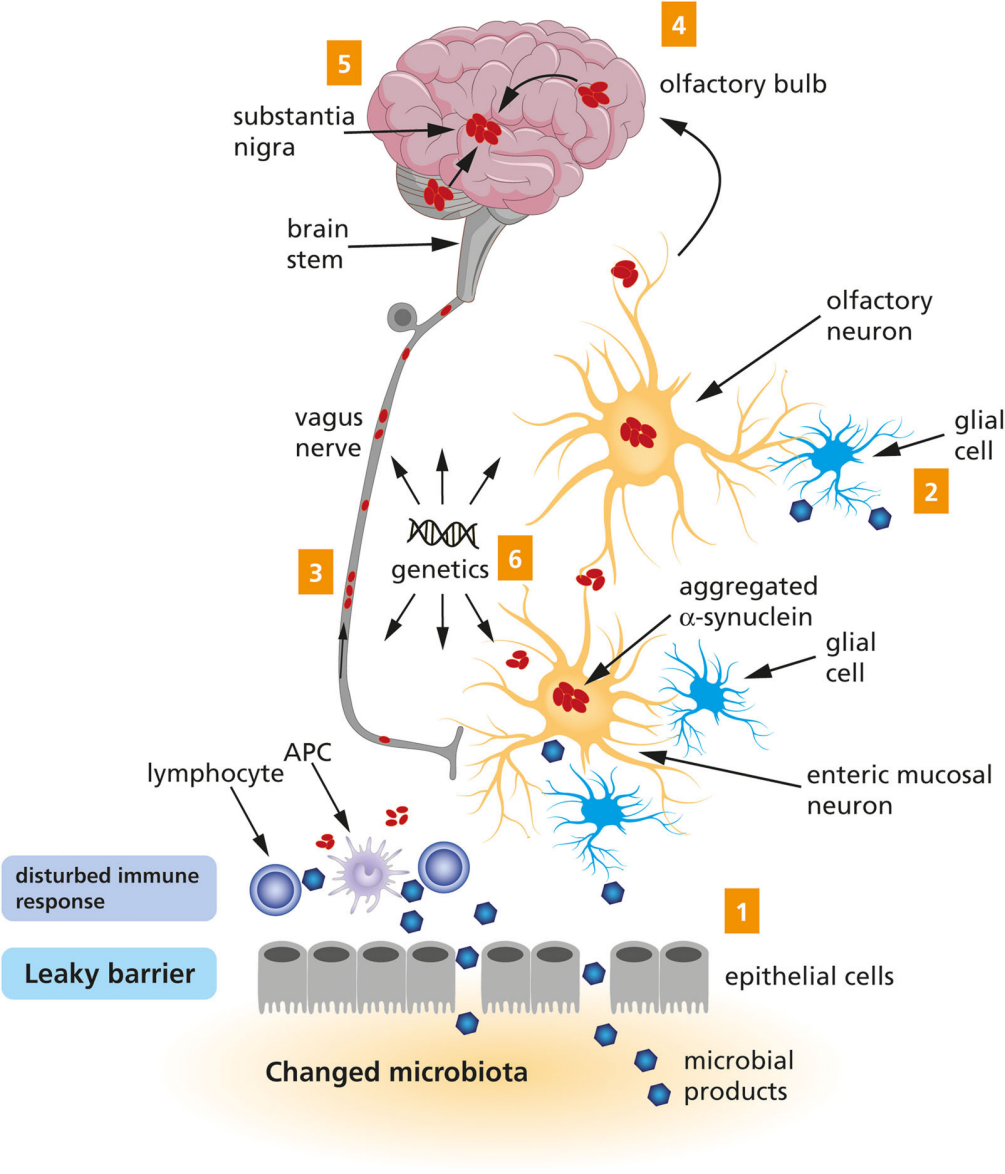
**A schematic representation of the Braak’s hypothesis of Parkinson’s disease (PD)**. Microbial products come into contact with olfactory and/or enteric neurons, which trigger the aggregation of α-Synuclein (1 and 2). The aggregated α-Synuclein spreads toward the central nervous system *via* the olfactory bulb and the vagus nerve (3 and 4). Eventually, the aggregated α-Synuclein arrives at the substantia nigra (5). Genetic factors are likely to contribute to PD, but the exact mechanism remains to be elucidated (6).

In 2003, Braak et al. postulated the hypothesis that an unknown pathogen (virus or bacterium) in the gut could be responsible for the initiation of sporadic PD (31), and they presented an associated staging system for PD based on a specific pattern of αSyn spreading (32). These publications were followed by the more encompassing dual-hit hypothesis, stating that sporadic PD starts in two places: the neurons of the nasal cavity and the neurons in the gut (33, 34). This is now known as Braak’s hypothesis. From these places, the pathology is hypothesized to spread according to a specific pattern, *via* the olfactory tract and the vagal nerve, respectively, toward and within the central nervous system (CNS). This process has been visualized in Figure 1. Interestingly, the hypothesized spread of disease to the spinal cord only takes place after the CNS has already become involved, and so the spinal cord is not considered to be a potential route for the spread of the disease from the periphery to the brain (33, 35).

### Preclinical and Clinical Evidence

There is experimental and clinical evidence supporting Braak’s hypothesis. Gastrointestinal problems like dysphagia, nausea, constipation and defecatory difficulty (36, 37), and the olfactory problem of the loss of smell (38) have been reported in PD. Additionally, the presence of LP in the neurons of the olfactory tract (39, 40) and the enteric nervous system (ENS) (41–43) has been confirmed. Severe LP in the ENS is positively correlated with constipation and motor problems in PD patients (44). There is also clinical evidence that LP in the nasal and gastrointestinal regions potentially precedes the diagnosis of the disease (32, 43, 45), leading to complaints of the digestive tract (46, 47) and problems with olfaction (48, 49) during the earlier stages of PD, before the onset of motor symptoms [this stage is also known as incidental Lewy body (LB) disease (50)].

In animal models, similar results have been found. Gastrointestinal problems have been described in models of advanced PD suffering from motor impairment (51–58), and in both genetic and toxin-induced models for earlier stages of PD without motor problems (59–61). Additionally, αSyn aggregations were found in the gastrointestinal tract of animal models of early (59, 60, 62) and advanced (51, 55) PD.

### Enteric Route: Clinical Evidence

From here on, this review will focus on the enteric route of Braak’s hypothesis. The importance of the ENS for PD is emphasized by circumstantial clinical evidence. The microbiome of control subjects contains a higher relative abundance of Prevotellaceae bacteria compared to PD patients, and within PD patients, a higher relative abundance of Enterobacteriaceae is associated with more postural and gait symptoms and less tremors (63). PD patients also suffer from increased inflammation in the colon, although colonic inflammation does not seem to be related to severity of gastrointestinal or motor problems (64). However, in PD patients, another sign of intestinal inflammation, an increased permeability of the intestinal barrier, seems to be related to increased staining in the intestinal mucosa for bacteria, oxidative stress, and αSyn (65). If changes in the microbiome predispose the (future) PD patient to a more pro-inflammatory environment in the intestines and increased barrier permeability, this could potentially lead to oxidative stress in the ENS. This oxidative stress could then trigger αSyn misfolding and aggregation, which could potentially spread from the ENS to the CNS, and eventually cause the hallmark motor problems. Therefore, changes in the microbiome and increased inflammation could directly negatively affect neurons of the ENS and be related to PD development, which is in accordance with Braak’s hypothesis.

Dietary components and dietary patterns have a considerable effect on the composition of the gut microbiome (66). The commensal gut microbiota thrive on the substrates that escape absorption in the small intestine and are available for colonic bacterial fermentation (67). For example, fiber-rich diets can enhance the growth of colonic bacteria that produce short-chain fatty acids (SCFA). These SCFA have systemic anti-inflammatory effects (68) and could therefore influence PD pathogenesis through this gut-mediated mechanism. Another example is Western diet (high in saturated fat and refined carbohydrates) that might result in dysbiotic microbiota (e.g., lower bifidobacteria, higher firmicutes, and proteobacteria) (69–71) and that could ultimately lead to a pro-inflammatory response and promote αSyn pathology. Therefore, it is essential to continue to research specific foods and dietary patterns that can improve gut health for PD risk reduction.

### Enteric Route: αSyn Spreading *via* Vagal Nerve

Another vital part of Braak’s hypothesis is the spread of αSyn pathology from the ENS to the CNS *via* the vagal nerve and the dorsal motor nucleus of the vagus (DMV) in the medulla oblongata, and the spread of pathology within the CNS from lower brainstem regions, toward the SN, and eventually the neocortex. Although these specific areas of the nervous system are affected by PD, certain neighboring areas seem to be spared, such as the nucleus tractus solitarius that is located next to and connected to the DMV. This indicates a non-uniform and specific pattern of the spreading of disease, which cannot be explained by the nearest neighbor rule (72). This specific pattern of spreading is supported by experimental and clinical evidence, although discussion about the validity of Braak’s hypothesis is still ongoing. In PD patients, LP has been found in the vagal nerve (73, 74) and the DMV (73, 75–78), and cell loss in the DMV of PD patients has also been reported (79). LP has been shown to occur in vagal nerves and DMV before it spreads to other parts of the CNS (32, 45, 76, 80), like the locus coeruleus and the SN, the mesocortex, the neocortex, and the prefrontal cortex (32). Additionally, truncal vagotomy might be associated with a decreased long-term risk of developing PD, which could be related to a hindrance of the spreading of disease *via* the vagal nerve, although this cannot yet be concluded from this single study (81). The spread of αSyn from the ENS to the CNS has also been studied in animal models. When the protein αSyn was injected in the wall of the stomach and duodenum of rats, it was able to spread through the vagal nerve to the DMV (82). Additionally, intragastric rotenone treatment of mice resulted in αSyn inclusions in the ENS, DMV, and SN, and cell loss in the SN (83). This rotenone-induced αSyn spreading could be stopped by vagotomy (84). These results show that the vagus nerve is involved in and essential for the spread of αSyn pathology from the ENS to the CNS in both rats and mice.

### Enteric Route: Spread of αSyn within CNS

Clinical evidence for the cellular transport of LP within the CNS comes from studies of PD patients whose grafts of fetal dopaminergic neurons showed LP and degeneration, indicating potential spread of pathology from host cells to graft cells (85–90). Host-to-graft transmission of αSyn has also been shown for mouse cortical neuronal stem cells (91) and mouse embryonic dopaminergic neurons (92) implanted in transgenic mice overexpressing human αSyn, and for rat embryonic dopaminergic neurons implanted in human αSyn overexpressing rats with (93) or without (94) striatal dopamine depletion. These results show that healthy neurons in the CNS are vulnerable to spread of disease by taking up LP from surrounding LP-affected neurons, although it does not indicate any specific pattern for this spreading.

### Transport of αSyn between Neurons

The ability of LP to spread through the nervous system raises the question what is the exact mechanism of transport of LP between neurons, and why the spread of LP follows a specific pattern, as suggested by Braak’s hypothesis. Both neuronal cell lines and primary neurons are able to excrete αSyn monomers, oligomers, and fibrils through unconventional calcium-dependent exocytosis from large dense core vesicles or *via* exosomes (84, 95–97). Once the αSyn is present in their environment, both neuronal cell lines and primary neurons seem to be able to take up free or exosome-bound fibrils and oligomers by endocytosis after which they are degraded in lysosomes (SH-SY5Y cells), while monomers seem to diffuse across the cell membrane and are not degraded (91, 97, 98). In a different study, the uptake was only found in proliferating SH-SY5Y neurons, but not in differentiated SH-SY5Y neurons, which could be due to the type of αSyn that was different from the other studies (radioactively labeled cell produced αSyn, versus different forms of recombinant human or non-human αSyn) (96). The transfer of specific αSyn molecules between cells of neuronal cell lines was proven in a coculture study of SH-SY5Y neurons expressing the same human αSyn labeled either green or red (92). Coculture resulted in double-labeled neurons, showing the process of subsequent excretion and uptake of αSyn by neighboring cells. After uptake, αSyn can be transported anterograde or retrograde through axons and passed on to other neurons (82, 84, 99–101), providing a potential highway for the spread of LP between connected nervous system regions in PD patients. A recent study shows that neuron-to-neuron αSyn transmission could be initiated by binding the transmembrane protein lymphocyte-activation gene 3 (LAG3). The study demonstrated that LAG3 binds αSyn preformed fibrils (PFFs) with high affinity and initiates αSyn PFF endocytosis, transmission, and toxicity in SH-SY5Y cells. Moreover, mice lacking LAG3 showed delayed αSyn PFF-induced pathology and reduced toxicity (102).

It is known that the neurons in the areas affected by LP in PD have specific characteristics that cause a high metabolic burden, which seems to make these neurons especially sensitive to oxidative stress and αSyn misfolding. These neurons have high levels of endogenous αSyn, they use monoamine neurotransmitters, have long and highly branched axons with no or poor myelination, and characteristic continuous activity patterns (72, 103, 104). Together this could explain why PD pathology develops in the specific pattern proposed by Braak, specifically affecting interconnected regions with vulnerable neurons like the DMV, while sparing neighboring areas like the nucleus tractus solitaries (72).

### Neurotoxicity of αSyn

It has been suggested that αSyn acts prion-like in PD. In this theory, pathologic, misfolded αSyn is an infectious protein spreading toxicity by forming a toxic template that seeds misfolding for nearby αSyn protein, turning the previously healthy protein into a toxic protein, causing LP. Excellent reviews on the prion-like theory of αSyn have been previously published (105, 106). The prion-like theory fits into Braak’s hypothesis, since the staging system of Braak is based on the regional presence (or absence) of LP and the spreading of LP, linking LP to severity of disease (32). The toxicity of αSyn in its different form is still undecided and remains the topic of many experiments, with one study reporting a cytoprotective function of αSyn aggregation (107), while others suggest that the oligomeric form of αSyn is the most toxic form of the protein (108–110). Foreign αSyn induces LP-resembling inclusion bodies in recipient neurons (91), caused by fibrils acting as exogenous seeds and recruiting endogenous αSyn into the inclusion body (92, 111), even in cells not overexpressing αSyn (101). Neuronal death resulting from αSyn exposure has also been shown (91), with a higher toxicity for oligomeric compared to monomeric species (96), and a higher toxicity of exosome bound oligomers compared to free oligomers (97). Inclusion bodies are linked to cell death, involving the loss of synaptic proteins and reduction in network connectivity (101).

In animal studies, injection of aggregated αSyn (derived from symptomatic transgenic mice) or synthetic αSyn fibrils into the brain of young, asymptomatic transgenic mice accelerated the formation and spread of αSyn inclusions throughout the brain resulted in early-onset motor symptoms, and reduced the lifespan of these mice (112, 113). Synthetic αSyn fibrils injected in the striatum also induced widespread LP, cell death of dopamine neurons in the SN, and motor deficits in wild-type mice (114). It has even been shown that fibril-seeded αSyn inclusions specifically increase neuronal death in αSyn transgenic mice in an experiment where neurons with or without inclusions were followed *in vivo*, providing direct evidence that αSyn inclusions were responsible for neuronal death (115). Injection of wild-type mice with patient-derived LB αSyn just above the SN resulted in degeneration of the dopamine fibers and cell bodies in the SN, and concomitant development of inclusion bodies exclusively consisting of endogenous αSyn, and reduced motor coordination and balance (116). Mice treated with non-LB αSyn (monomers) did not develop these lesions. Similar results were found in rhesus monkeys; injection of patient-derived LB αSyn in the striatum or SN resulted in reduced nigrostriatal dopaminergic innervation, increased αSyn immunoreactivity in connected brain regions after striatal injection (but not after SN injection), without LP or motor symptoms (116). Taken together, these results do not definitively confirm or reject the prion-like theory in the context of Braak’s hypothesis. However, a picture emerges where αSyn oligomers are likely toxic to neurons, and inclusion bodies are linked to neuronal death, which might or might not lead to motor symptoms. Although the studies included here were performed in the CNS, the emerging picture of oligomer toxicity and inclusion body-induced neuronal death could also be applicable to the ENS and other parts of the peripheral nervous system.

## Criticism to Braak’s Hypothesis

### Criticism to the Specific Pattern of Spreading

Despite the *in vitro, in vivo*, and clinical support for Braak’s hypothesis, there is also doubt whether it accurately describes the development of PD in all patients (117, 118). A large subset of 51–83% of PD patients follow Braak’s staging, while a smaller subset of 7–11% do not have LP in the DMV while higher brain regions are affected (119–124). Additionally, there is no correlation between severity of LP in the DMV and in the limbic system or neocortex (125). Also, LP in the ENS is not correlated to olfactory problems, and 27–33% of PD patients did not show any LP in the ENS, which does not support the dual-hit hypothesis (64, 126), although it is known that LP can be restricted to the olfactory system in the early stage of the disease (124). Additionally, people with incidental LB disease seem to have a similar distribution but milder expression of LP compared to PD patients (50, 127) and can show LP in the SN and other areas of the brain without LP or neuronal loss in the DMV (77, 122, 128, 129) or LP in the vagus nerve (45), favoring multiple origination sites for LP instead of a spread from ENS to CNS *via* the vagus nerve. Additionally, Braak’s hypothesis does not explain how or why cardiac sympathetic nerves are affected in early PD (129). Therefore, it seems safe to conclude that not all PD patients adhere to the specific pattern of LP spread proposed by Braak.

### Criticism to the Link between LP, Neuronal Loss, and PD Symptoms

Other studies have shown that the link between LP and clinical PD symptoms should be questioned. Only 45% of people with widespread LP in the brain are diagnosed with dementia or motor symptoms (121) and only about 10% of people with LP in the SN, DMV, and/or basal forebrain are diagnosed with PD (130). Additionally, neurodegeneration in the SN might precede LP (131). Therefore, the spreading of LP, whether according to Braak’s staging system or not, might not be as tightly bound to clinical symptoms as has been suggested by Braak.

The basic science underlying Braak’s hypothesis has also been questioned (118, 132), because in the initial studies all cases were preselected for LP in the DMV (32, 76), systematically excluding any cases where LP in higher brain regions was found in the absence of LP in the DMV, which seems to have led to a selection bias and the inclusion of non-representative samples in the preclinical PD group in the original research (132). The limited clinical information on the preclinical PD group and the absence of information on neuronal cell loss in the original Braak papers have also been criticized (117, 118, 132). It has been suggested that neuronal loss and activation of glial cells should be part of future pathological analysis of PD to better describe disease progression, since the clinical significance of LP is not yet clear and might be less important than previously thought (121, 130, 131).

### Studying Neuronal Loss and Glial Activation in Future PD Research

Studying neuronal loss together with LP during PD development is important because neuronal loss in the SN shows a linear relationship with motor symptoms (133), while LP in the overall brain only shows a trend for positive correlation with motor symptoms (124). Additionally, LP is not related to dopaminergic cell loss in the striatum (124), and may (124) or may not (134) be related to dopaminergic cell loss in the SN of PD patients. Therefore, it can be concluded that neuronal loss and LP are not interchangeable hallmarks for PD progression or severity of disease, but should rather be seen as complimentary to each other.

Studying the activation of glial cells is important because neuroinflammation is an important factor in PD development, and glial cells are major contributors to neuroinflammation, partially through toll-like receptors (TLRs) (22–27). Especially TLR2 and -4 are important in PD, since their expression is increased in the brain of PD patients, and a polymorphism resulting in lower expression of TLR2 tends to be linked to an increased risk of PD (135–138). Preclinical research has confirmed the importance of TLR2 and -4 for PD and has specifically shown their importance in the context of glial-induced inflammation and αSyn uptake by glial cells (138–149).

## Conclusion

Reviewing the current literature it can be concluded that there is much evidence to support Braak’s hypothesis. Enteric and olfactory pathology and dysfunction are well-known characteristics of early and late PD. The vagus nerve and DMV form a likely route for αSyn pathology to spread from the ENS to the CNS, and αSyn is able to spread cellularly within the CNS. Neurons are able to transmit different forms of αSyn protein to each other and to transport αSyn *via* their axons, which enables the spread of the potentially toxic oligomeric variety of the protein, which could be the basic mechanism underlying the specific pattern of LP spread in PD as proposed by Braak. It then seems possible that a pathogen or environmental toxin might provoke local inflammation and oxidative stress in the gut, thereby initiating αSyn deposition that is subsequently disseminated to the CNS. Hypothetically, the toxic αSyn can lead to neuronal death. (Micro)glial cells and surviving neurons can then be activated through the release of danger associated molecular patterns and subsequent activation of TLRs. This would trigger a vicious circle of neuroinflammation.

However, it can also be concluded that a significant portion of PD patients do not follow Braak’s staging system. It has been discovered that a subgroup of levodopa-responsive PD patients who develop PD at a young age and have a long duration clinical course with predominantly motor symptoms, and dementia only at the later stages, seem to follow Braak’s staging, while other levodopa-responsive PD patients did not (80). In addition to this, a LB staging system has been proposed, which encompasses all patient groups, a system wherein LP staging correlates well with motor symptoms and cognitive decline (124), and allowing for patients who show a spread of LP not accounted for in Braak’s hypothesis. Unfortunately, the staging system is only describing the different observed patterns of LP spread, while not answering the question as to the cause of the non-Braak patterns. What is the reason or explanation for these other types of patterns to occur? This question remains to be answered.

We conclude that Braak’s hypothesis and the Braak staging system are valuable and useful for the future study of PD, and these theories are likely to accurately describe disease initiation and progression in a subgroup of PD patients with young onset and long duration of disease. However, a similar theory describing the initiation and disease progression in other PD patients is still sorely lacking and deserves to be elucidated. To better understand the progression of LP and PD in different patient groups, it is necessary to study people longitudinally during disease development, and especially in the earliest stages of PD. This should lead to a larger theory describing different disease processes, all leading to PD, including Braak’s hypothesis. This theory could offer useful insight into specific targets for disease prevention or disease treatment, dependent on the type of LP disease the patient is likely suffering from. Either more optimal treatment with currently available drugs and technology, or the development of new treatments could be the result.

## Author Contributions

CR, PP-P, JG, RW, and AK conceived and designed the review. CR wrote the first draft of the review with PP-P’s assistance. JG, RW, and AK reviewed and critiqued the manuscript. All the authors were responsible for the decision to submit the manuscript for publication.

## Conflict of Interest Statement

JG is an employee of Nutricia Research, Utrecht, The Netherlands. All other authors report no potential conflicts of interest.
